# Case of Paralysis

**Published:** 1856-06

**Authors:** S. O. Long

**Affiliations:** Big Rock, Illinois


ARTICLE III.—*Case of Paralysis.* By S. O. LONG, of Big Rock, Illinois.

Mr. B., a worthy and industrious man, though of somewhat intemperate habits, in returning to his home, May 10th, 1854, fell from his wagon, his back, in the fall, striking the wheel; and, on his attempting to rise, found himself perfectly helpless, and unable to move, excepting his head and arms. Was carried to his house by some of the neighbors, and I was called to see him the same night.

*Present Condition.* Complained of considerable pain between the shoulders and through the chest. In passing my hand over the spinous process, there was some tenderness about the fifth and sixth dorsal. Pulse somewhat accelerated—tongue partly covered with whitish coat—moderate thirst. Laying my hand on the abdomen produced no sensation at all, neither in the limbs. In fact, all motion and sensation were entirely lost. Perfect paraplegia of lower part of the body and extremities—the abdomen hard and tympanitic—bladder distended, with inability to pass his water—bowels constipated. I directed fomentations to be applied to the dorsal region. Introduced a catheter, and evacuated a large quantity of urine. The bowels to be opened with castor oil, ℥ss.; turpentine, ʒi.; repeated every two hours, if necessary: then give Dover, grs. viii., every four hours; and nitrate potash, grs. v., and tart. emet., 1-10 gr., between; with morphine, $\frac{1}{8}$ gr., every hour, till pain is controlled.

12th. Dr. C. Wheeler visited him in consultation with me. The fomentation seemed to afford him considerable comfort, and to some extent relieved the tenderness. Oil and turpentine acted on the bowels freely, though did not remove the tympanitis. Pulse less frequent—skin moist—tongue less coated and moist—unable to pass water. Dr. W. advised cupping between the shoulders, and apply warm or cold water to the part, as most agreeable to the patient; evacuating the bladder with the catheter, and light diet. I applied a cupping glass, after scarifying, and took ℥ vi.; which, as he said, "made him feel as though he could get up and walk."

13th. Continued the treatment.

14th. No change for the better—bowels torpid—moved them with stimulating enema and turpentine. Bladder distended—attempted to relieve with the catheter, introduced without any difficulty, but no urine passed. Naturally presuming that it had not entered the bladder, I partly withdrew, and again gently pressed, and could feel it pass into the neck, and still no water followed. I then exchanged for another instrument, and made another attempt, with no better success. On withdrawing and examining, I found the eye clogged with a bloody, jelly-like substance. I then enlarged the eye of the catheter, and, with a syringe, injected some warm water through the catheter into the bladder; but nothing returned. After some hours, Dr. Allaire visited him in consultation, and, with a different instrument, drew off a large quantity of stale urine.

Now, in regard to this difficulty in evacuating the urine, I could not, and have not exactly satisfied myself, though Dr. Allaire was of the opinion that it was caused by the stricture, which I did not pass. It may be so, as the result would seem to justify. The treatment agreed upon, was to apply a blister between the shoulders and one side of the spinal column, and when partly healed, to repeat to the other side; try the effects of tinct. canths., for the palsy of bladder—that failing, then strychnine, in 1-12 gr. doses, three times a day; animal food, with friction to the lower limbs; keeping the bowels open, and catheterism. This course was carried on until the 24th, without any improvement in the symptoms. At this time, he began to be afflicted with severe spasms, which were almost immediately controlled for the time by inhalations of chloroform, ℨii. to ℨiv.; which, however, would arrest the action of the heart, cause congestion of the lungs, and aphyxia, to such a degree, that I thought best to discontinue it—in fact, he refused to inhale more. I them prescribed morphine, in $\frac{1}{8}$ to $\frac{1}{4}$ gr. doses, to repeat every hour, till the spasms were controlled, or the system brought under its influence. This treatment would nearly allay the spasm, but it required such a quantity, in the aggregate, as to produce narcosis. Belladonna was given till it acted on the system; without, however, any alleviation of the complaint. I now proposed, as the blistered surface was healed, to insert a seton near the injured part, as a means of allaying the chronic inflammation and promoting absorption. He would not consent to this course on any condition, neither mercury in any form. Continued the treatment as given above, together with anodynes, and wrote a line to Dr. Allaire upon the case; and received from him an interesting communication upon the subject, expressing his views of the case. As the course he proposed would be judicious in similar cases, I cannot do better than to insert an extract from his letter:—

"It is certain that the cause of all the symptoms, is the local injury and its effects. The effects of the injury are, probably, thickening of the fibrous tissues surrounding the spinal cord at the part, and perhaps, also, serous effusion around and softening of the cord. The indication, then, is to reduce what there is of chronic inflammation in the part, and promote the absorption of thickened tissues. I believe this can be best done by putting him under the influence of mercury, and inserting an issue or seton near the part. As a temporary palliation of the spasms, you might try ext. stramonium, giving a dose three times a day; but opium, in some form, probably, will have to be your main dependence. If possible, I should try to get along by giving only at night, and in doses large enough to produce sleep, thus imitating nature. The mercury, when I have to give it a long time, as you may in this case, I use as follows: give 1-20 of a grain of calomel every hour, until the gums are touched. Then give the same dose morning and night. If this did not keep up a gentle influence, then repeat the dose."

I stated to Mr. B., Dr. Allaire's proposed course of treating the case; and, although he had confidence in his professional abilities, declined any further treatment, and said he had concluded to supply himself with and make use of Dr. Jayne's medicines, believing they would soon "get him on his feet again." He run through the list of his medicines in a few weeks, and then omitted them entirely.

*Sept.* 19th. I was requested to visit him and insert a seton, which I did, though without expecting a beneficial result—he gradually failing. Soon after, dysentery supervened, which, together with local injury, produced such a condition that death in a few days relieved him of suffering.

